# Efficacy of Acupuncture on Quality of Life, Functional Performance, Dyspnea, and Pulmonary Function in Patients with Chronic Obstructive Pulmonary Disease: Protocol for a Randomized Clinical Trial

**DOI:** 10.3390/jcm11113048

**Published:** 2022-05-28

**Authors:** Renato Fleury Cardoso, Ana Cristina Rodrigues Lacerda, Vanessa Pereira Lima, Lucas Fróis Fernandes de Oliveira, Sofia Fróis Fernandes de Oliveira, Rafaela Paula Araújo, Cecylia Leiber Fernandes e Castro, Flávia Pereira da Silva, Lizânia Vieira de Paiva, Lia Dietrich, Pedro Henrique Scheidt Figueiredo, Henrique Silveira Costa, Mario Bernardo-Filho, Danúbia da Cunha de Sá-Caputo, Vanessa Amaral Mendonça, Redha Taiar

**Affiliations:** 1 Postgraduate Program in Health Sciences (PPGCS), Federal University of Jequitinhonha and Mucuri Valleys (UFVJM), Diamantina 39803-371, Brazil; cardoso.renato@ufvjm.edu.br (R.F.C.); lacerda.acr@ufvjm.edu.br (A.C.R.L.); vaafisio@hotmail.com (V.A.M.); 2 Physiotherapy Department, Federal University of Jequitinhonha and Mucuri Valleys, Diamantina 39803-371, Brazil; vanessa.lima@ufvjm.edu.br (V.P.L.); lucaasfrois@hotmail.com (L.F.F.d.O.); sofiafroisf@hotmail.com (S.F.F.d.O.); rafaela.araujo@ufvjm.edu.br (R.P.A.); cecylialeiber@hotmail.com (C.L.F.e.C.); flavia.silva@ufvjm.edu.br (F.P.d.S.); pedro.figueiredo@ufvjm.edu.br (P.H.S.F.), henriquesilveira@yahoo.com.br (H.S.C.); 3 Postgraduate Program in Health, Society and Environment (PPGSASA), Federal University of Jequitinhonha and Mucuri Valleys (UFVJM), Diamantina 39803-371, Brazil; lizania.paiva@ufvjm.edu.br; 4 Dentistry Department, Federal University of Jequitinhonha and Mucuri Valleys, Diamantina 39803-371, Brazil; lia.dietrich@ufvjm.edu.br; 5 Laboratory of Mechanical Vibrations and Integrative Practices, State University of Rio de Janeiro, Rio de Janeiro 20550-013, Brazil; bernardofilhom@gmail.com (M.B.-F.); dradanubia@gmail.com (D.d.C.d.S.-C.); 6 MATériaux et Ingénierie Mécanique (MATIM), Université de Reims Champagne-Ardenne, 51100 Reims, France

**Keywords:** chronic obstructive pulmonary disease, COPD, acupuncture, acupuncture therapy, acupuncture treatment, quality of life, health-related quality of life

## Abstract

Chronic obstructive pulmonary disease (COPD) is a respiratory disease characterized by the presence of chronic airflow obstruction. Previous studies have evaluated the effect of acupuncture treatment (AT) in patients with COPD. Nevertheless, these studies show a great deal of heterogeneity in treatment protocols, having sample sizes that are too small to estimate and clarify effect size and heterogeneity in patients’ baseline. The aim of this study is to evaluate the effectiveness of acupuncture on quality of life, functional performance, dyspnea, and pulmonary function in patients with COPD. As such, patients will go through the following three phases: Phase I–pretreatment: period of subject selection and inclusion in the protocol, with an interview and performance of exams and tests as follows: Mini-Cog, dual-energy X-ray absorptiometry, spirometry, the Patient-Generated Index, Saint George’s Respiratory Questionnaire, the six-minute walk test, the London Chest Activity of Daily Living, and the COPD Assessment Test. Phase II–8 weeks of treatment, with AT 3 times a week, with two parallel groups: Group I–with 50 subjects–AT according to the recommended technical standards; Group II–with 50 subjects–Control, without acupuncture. Phase III–Continuation of AT for 8 weeks, maintaining the subjects in the previously allocated groups and following the same methodology.

## 1. Introduction

Chronic obstructive pulmonary disease (COPD) is a preventable and treatable respiratory disease characterized by the presence of chronic airflow obstruction that is not fully reversible [1] and by the presence of systemic oxidative stress and inflammation biomarkers [2,3]. Studies on the prevalence of COPD suggest that around a quarter (¼) of adults aged 40 years and older have moderate airflow obstruction [4].

Although COPD compromises the lungs, it also has significant systemic consequences [1,4,5,6]. COPD patients often adopt a sedentary lifestyle that can precipitate the onset of muscle deconditioning through inactivity and a cycle of clinical deterioration [1,7,8,9], negatively impacting the quality of life of patients with COPD [1,10]. Thus, although these patients maintain activities of daily living (ADL) related to self-care, mobility, food, and personal hygiene, their ADLs are considered to be of lesser intensity when compared to healthy individuals [11]. The same is true for physical activity as COPD patients often maintain lower levels of physical activity, according to international guidelines for maintaining physical health [7,8].

The concept of quality of life (QoL) refers to objective and, to a greater extent, subjective indicators of happiness and satisfaction [12]. According to the World Health Organization, QoL is defined as “an individual’s perception of their position in life in the context of the culture and value system in which they live, and in relation to their goals, expectations, standards, and concerns” [13]. In this sense, the administration of questionnaires to assess the HRQoL of patients with COPD has been widely used, generating reliable, valid, and reproducible evidence [14,15].

The complementary role of acupuncture in alleviating the symptoms of various diseases has become increasingly relevant [16]. It is currently accepted that the stimulation of acupuncture points causes the release of neurotransmitters in the central nervous system, in addition to other substances responsible for the responses promoting analgesia, restoration of organic functions, and immune modulation. The World Health Organization recommends acupuncture to its member states, having produced several publications on its efficacy and safety, training of professionals, research methods, and evaluation of the therapeutic results of complementary and traditional medicines [17,18]. In addition, the consensus of the National Institutes of Health of the United States endorsed the indication of acupuncture, alone or as an adjunct, in several chronic conditions including COPD [19]. Additionally, a portion of COPD patients do not respond significantly to pulmonary rehabilitation, and therefore require alternative forms of intervention [20].

Previous studies have evaluated the effect of acupuncture treatment (AT) on COPD patients [21,22], but recent systematic reviews demonstrate great heterogeneity in treatment protocols, including different types and numbers of points of acupuncture, treatment regimens, treatment durations, inappropriate sample size to estimate and clarify effect size, and heterogeneity in the main outcomes of patients evaluated at baseline and in treatments received, demonstrating low methodological quality [23,24]. In addition, there are also studies that evaluate the additive effect [21] and studies that compare AT with certain other interventions [25], leaving a gap regarding the isolated effect of AT. This study hypothesizes that AT will result in an improvement in the quality of life (primary outcome) of patients with COPD, in addition to an improvement in functional performance and pulmonary function, promoting a reduction in the impact of the disease on the lives of such patients.

## 2. Materials and Methods

### 2.1. Design

The study will be a two-arm, parallel-group, prospective, computer-randomized, controlled, evaluator-blind design lasting for 16 weeks (two eight-week intervention periods). The study was registered on the www.ensaiosclinicos.gov.br (accessed on 23 May 2022) (REBEC) website. The protocol has been developed according to the SPIRIT guidelines [26] and described according to the CONSORT statements [27]. The acupuncture protocol will follow the recommendations of the Standards for Reporting Interventions in Clinical Trials of Acupuncture (STRICTA), a formal extension of CONSORT. STRICTA was designed to improve the integrity and transparency of reporting interventions in clinical trials of acupuncture so that such trials can be more accurately interpreted and easily replicated [28]. Furthermore, the intervention will be reported according to the Model for Intervention Description and Replication (TIDieR) checklist and guide [29].

### 2.2. Patients

Patients with a clinical diagnosis of COPD who meet the inclusion criteria will be recruited from the waiting list of the Federal University of the Jequitinhonha and Mucuri Valleys (UFVJM) Physiotherapy school clinic, as well as in doctors’ offices and hospitals and through pamphlets and advertisements on local radio.

Patients will be aware that they will be randomized into one of two groups. Group I will receive AT and Group II will be without AT–we chose a control group without any intervention in order to analyze the isolated effect of AT. At the end of the project, if the results are favorable, patients in the control group will be offered the possibility of receiving AT.

The sample size was estimated using the GPower^®^ program (Franz Faul, Universitat Kiel, Kiel, Germany), version 3.1.9.2. For this, a priori analysis was used, considering the t-test for comparisons between groups, for the quality-of-life variable evaluated by the Saint George Respiratory Questionnaire (SGRQ)-Total score [22]. Thus, considering an effect size of 0.59 [22], power of 0.80%, and alpha error of 5%, the sample size was estimated at 50 patients for each group, totaling 100 patients. These calculations assumed a 20% loss in the worst-case scenario.

### 2.3. Randomization

Randomization will be generated by a website (https://www.randomizer.org/ (accessed on 23 May 2022)) and performed by a researcher not involved in the recruitment, treatment, or evaluation of patients. This investigator will be instructed not to disclose the scheduled intervention to the other investigators until the completion of the study. Since randomization will occur after the initial evaluation, it is characterized as a blind distribution. The sequence will be generated in blocks of four patients in random order. The allocation will be hidden in numerical sequence, in opaque sealed envelopes.

### 2.4. Eligibility

#### 2.4.1. Inclusion Criteria

- Clinical diagnosis of COPD according to GOLD [1].
- Ability to offer written authorization or nominate a person to read the Statement of Free and Informed Consent, giving the research patient’s agreement.
- Patients over 65 years of age with preserved cognitive function according to the Mini-Cog, that is, those who scored at least 3 to 5 points [30].
- Absence of exacerbation and stability of drug treatment in the last month.
- A 3-month absence from participation in a pulmonary rehabilitation program.

#### 2.4.2. Exclusion Criteria

- Patient with a previous medical diagnosis of a disease that affects cognitive function and inhibits understanding of the questionnaires.
- Patients unable to perform any of the assessments.
- Previous acupuncture therapy.
- Being, or having been, in a rehabilitation group in the last 3 months before starting the protocol.
- Patients who present an exacerbation of the clinical condition during the collection period will be excluded.

### 2.5. Intervention

The tests and questionnaires applied after phases II and III will be carried out within 48 h after the last session.

**Figure 1:**
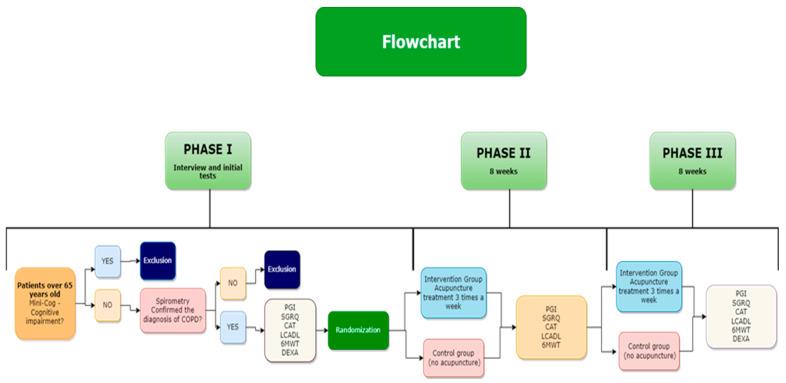
Flow of participants throughout in all stages of the research.

The period for carrying out this protocol will be from 2022 to 2025. Figure 1 presents the flow chart of the full study protocol.

#### Acupuncture

Acupuncture needles made of stainless steel, 0.25 mm gauge and 3 cm long, approved by the Brazilian National Health Surveillance Agency (ANVISA) will be used.

The description of the technique and methodology follows the principles suggested by the Standards for Reporting Interventions in Clinical Trials of Acupuncture (STRICTA) [28], described below:

(a) the Traditional Chinese Medicine needling style will be used, based on the literature in the area and clinical consensus.
(b) treatment reasoning will be based on Chinese acupuncture literature, consensual methods, and literary references.
(c) the selection of needling points will not change throughout the treatment.
(d) the needling will be manual.

After the insertion of needles, manipulation will be carried out with adequate stimulus to obtain Dé Qi (acupuncture sensation) at determined and preestablished points for all patients.

The procedure for each session will be as follows:

Patients in the supine position will have the needles removed 30 min after the last needle is inserted.

The following acupoints will be used:

- Zusanli-stomach 36 (ST36 bilateral).
- Xuehai-spleen 10 (SP10 bilateral)
- Qihai-Ren Mai 6 (RM6 or CV6).
- Danzhong–Ren Mai 17 (RM17 or CV17).
- Chize-lung 5 (LU5 bilateral).
- Lieque-lung 7 (LU7 bilateral).

A total of 10 needles will be used per session.

After a thorough evaluation (Hsieh and colleagues, 2020) of the acupoints used in prior randomized clinical trial studies for COPD therapy, the points were chosen. Furthermore, sites were chosen that allows the patient to remain supine during treatment without changing positions [31].

The order of puncture will follow the traditional concept of needling. Because the problem (COPD) is at the top (lungs), the puncturing starts from the bottom. Thus, the order of puncture will follow the sequence: ST36, SP10, RM6, RM17, LU5, and LU7.

### 2.6. Outcome Measures

The primary outcome (quality of life) will be assessed using the SGRQ and PGI questionnaires. Secondary outcomes will be evaluated using DEXA, Spirometry, 6MWT, LCADL, and CAT.

#### 2.6.1. Questionnaires, Scales and Tests

##### Mini-Cog

The Mini-Cog will be applied only in the first phase of the experiment, as an inclusion criterion for patients over 65 years of age. The test consists of a three-word memorization task and a clock drawing task. The individual must be able to remember the three words after making the drawing, scoring 1 point for each word they remember and 2 points for correctly drawing the clock [30,32].

The Mini-Cog has a total score of 5 points. Results ranging from 3 to 5 points are considered normal [32].

##### Double Energy X-ray Absorptiometry (DEXA)

Total body mass, fat mass, lean mass, bone mineral density, and height will be evaluated using dual-energy radiological absorbance (DEXA) (Lunar Radiation Corporation, Madison, WI, USA, DPX model). Fat mass and lean mass will be evaluated by total body analysis and by body segment (upper, lower and trunk). For this analysis, patients will be positioned in the scanning area of the equipment so that the sagittal line marked on the equipment passes under the center of certain anatomical points such as the skull, spine, pelvis, and lower limbs. Patients will be evaluated wearing light clothes, without the use of any metal object that could interfere with the measurements.

DEXA will be used to characterize the sample and to assess changes in the subjects’ body composition. Therefore, it will be evaluated in Phase I and at the end of Phase III.

##### Spirometry

Spirometry is the measurement of the air that enters and leaves the lungs [33,34]. Spirometry is a test that enables the diagnosis and quantification of ventilatory disorders. It will be used to assess the individual’s pulmonary function and characterize the studied sample according to the GOLD criteria [1].

Spirometry will be performed using the MIR Spirometer, Model Minispir, manufactured by MIR Medical International Research (Rome, Italy), with the individual sitting with trunk support, with arms along the body and wearing a nose clip. The subject will be asked to place the mouthpiece above the tongue and close the mouth tightly around it; at the researcher’s command, the individual will make a deep inspiration until total lung capacity (TLC) has been reached, followed by a “fast and prolonged” forced expiration, until the examiner’s end signal, when the patient will make another maximum inspiration with the mouthpiece still in the mouth. At least three measurements will be performed, three of which are acceptable and two reproducible, for evaluation; the best values will be recorded according to the 1st Brazilian Consensus on Spirometry. The test will analyze forced vital capacity (FVC); forced expiratory volume in the first second (FEV1); forced expiratory flows at 25%, 75%, and 25–75% of the FVC curve; peak expiratory flow (PEF); the relationship between FEV1 and FVC (FEV1/FVC); and inspiratory capacity (IC), which is the amount of air that can be inhaled from resting expiratory level to TLC. These parameters will be expressed in absolute values and percentage of the predicted value, according to the GOLD reference values [1].

The spirometer will be calibrated each day before the tests. Calibration will involve measuring the output of the spirometer and the sensitivity of the recording device or generating a software correction factor, and it will therefore involve tuning the equipment for performance within certain limits. For this purpose, a 3 L syringe supplied by the manufacturer will be used along with the equipment.

Spirometry will be applied in Phase I to confirm the diagnosis according to GOLD and characterize the sample, and at the end of Phase II and Phase III to evaluate the response to the acupuncture intervention.

##### Patient-Generated Index (PGI)

The PGI is completed in three steps: (1) Patients identify the five most important areas of their life affected by the disease, in this case, chronic obstructive pulmonary disease; (2) patients rate how much each area was affected by COPD using a scale of 0 to 6, where 0 is the worst imaginable and 6 is exactly how they would like it to be; (3) patients now imagine they have 10 “tokens” to spend on improving selected areas and allocate these tokens to areas according to their priority. An overall index is then calculated by multiplying the ratings for each area in Step 2 by the proportion of tokens given to that area in Step 3, which are then added to produce an index where higher scores indicate higher QoL [14].

It is worth mentioning that for the PGI there is a minimum detectable difference of 10.8 [14].

The PGI will be used to assess QoL, which will be analyzed in Phase I and at the ends of Phase II and Phase III.

##### Saint George’s Respiratory Questionnaire (SGRQ)

The SGRQ addresses aspects related to three domains: symptoms, activity, and psychosocial impacts that respiratory disease inflicts on the patient. Each domain has a maximum possible score; the points for each answer are added up and the total is referred to as a percentage of the maximum. A total score is calculated from 0 (no health impairment) to 100 (maximum health impairment). In addition to the total score, there is also a score for each domain of symptoms, activity, and impact, which are also scored from 0 to 100. Values above 10% reflect an altered QOL in that domain. Changes equal to or greater than 4% after an intervention, in any domain or the total number of points, indicate a significant change in the patients’ QoL [35].

The SGRQ will be used to assess QoL, which will be analyzed in Phase I and at the ends of Phase II and Phase III.

##### London Chest Activity of Daily Living (LCADL)

The London Chest Activity of Daily Living (LCADL) scale has four domains (personal care, household activities, physical activities, and leisure activities), with 15 questions in total, to assess the limitations to ADLs in patients with COPD [36]. Each question in the domains receives a score from 0 to 5, indicated by the patient, with the highest value representing the maximum inability to perform ADL due to dyspnea. The total score can range from 0 to 75 points, and the higher the value, the greater the limitation in ADL. The scale also has question 16, which refers to a specification of ADL impairment due to dyspnea, in any situation, and the patient must answer this question by ticking one of the three alternatives: “a lot”, “little”, or “not at all” [37].

The LCADL will be used to assess ADLs, which will be analyzed in Phase I and at the ends of Phase II and Phase III.

##### Six-Minute Walk Test (6MWT)

To evaluate functional performance, the 6MWT will also be applied according to the recommendations of the American Thoracic Society [38]. The test will be performed in a 30 m corridor, on a flat surface on which the subjects will be instructed to walk as fast as possible, without running, for six minutes. The subject will be allowed to rest during the test and resume walking as soon as they are able. During the walk, the examiner will give verbal stimuli every minute, always using the same tone of voice and the standardized phrase: “you are doing very well”. The subject will be instructed to interrupt the test if he presents pain in the lower limbs, tachycardia, dizziness, or any other symptom of discomfort. Immediately before and after the test, systemic blood pressure (BP), heart rate (HR), oxygen saturation (SpO2), and level of dyspnea and lower limb fatigue (Borg scale) will be measured. Two tests will be performed with an interval of 30 min between them, and the test in which the patient has walked the longest distance will be used for analysis. If the difference in the distance walked between the two tests is greater than 10%, a third test will be performed [38,39]. It is noteworthy that a mean increase of 30 m in 6MWT covered distance after pulmonary rehabilitation in COPD patients indicates a minimal clinically important increase [39,40].

The 6MWT will be used to evaluate functional performance; therefore, it will be evaluated in Phase I and at the ends of Phase II and Phase III.

##### COPD Assessment Test (CAT)

The CAT is composed of eight items, called cough, phlegm, chest tightness, shortness of breath, limitations in household activities, confidence in leaving home, sleep, and energy. For each item, the patient chooses only one answer option, the score of which varies from zero to five. At the end of the test, all response scores are added up, and the clinical impact of COPD is evaluated according to the stratification score of the CAT development and validation study [41]. The results vary according to the range of scores obtained, being classified for clinical impact as follows: 6–10 points, mild; 11–20, moderate; 21–30, severe; and 31–40, very severe [42].

The CAT will be used to assess the impact of COPD on patients’ lives, which will be analyzed in Phase I and at the ends of Phase II and Phase III.

### 2.7. Ethics

Each patient will receive information about the purpose and structure of the research and written consent will be required through the Free and Informed Consent Form (ICF) to participate in the study. Patients may opt out of the study at any time before or during implementation of the protocol.

The project has been approved by the Ethics and Research Committee of the Federal University of Jequitinhonha and Mucuri Valleys (reference number 5.274.273).

### 2.8. Analyses

Statistical analysis will be performed following the intention-to-treat principle. Data will be analyzed using the SPSS Statistics statistical package (v.22.0; IBM Corp, Armonk, NY, USA). The normality of the data will be verified using the Kolmogorov-Smirnov test and the homoscedasticity of the data through the Levene test. The parametric data will be expressed as mean and standard deviation. In the case of nonparametric data, the median and its upper and lower limits will be expressed. The data will be analyzed using generalized linear mixed-effects models for repeated measures with post hoc Bonferroni analysis for correction. For all tests, an alpha of 5% will be adopted. Effect sizes will be interpreted based on their minimal clinically important differences (MCIDs).

#### Treatment of Lost Data (Missing Data)

Missing data will be classified as missed and nonrandom (PNA) when dropouts are due to a lack of efficacy and adverse effects and as missed completely by chance (PCA) when a loss to follow-up does not depend on observed or unobserved measures (e.g., patient moves to another city for non-health reasons). We are planning to perform mixed-effects models for repeated measures (repeated-measures ANOVA) to deal with missing data due to PCA and use single imputation methods (such as best- or worst-case imputation, i.e., assigning the worst possible value of the result for dropouts for a negative reason–treatment failure and the best possible value for positive dropouts (cures)) when considering the absence a PNA. We are planning sensitivity analyses to assess whether the methods used to deal with missing data produce any important differences in the results [43].

## 3. Discussion

Previous studies on acupuncture for the treatment of COPD demonstrate that acupuncture can result in important clinical improvements in quality of life and dyspnea [21,44]. However, existing studies provide insufficient evidence due to methodological limitations, including poor study design, inadequate control groups, and variations in AT. A systematic review on the topic published in 2018 showed that the methodological quality of the included studies was generally low. For example, most of the included studies were at high risk of performance bias. In addition, most analyses of the data in the meta-analysis indicated heterogeneity across studies. Finally, there were several forms of intervention adjunct to acupuncture, which makes it difficult to assess the effectiveness of acupuncture alone [45].

Therefore, more rigorously designed studies are needed to elucidate the efficacy of acupuncture therapy for the treatment of patients with COPD and its response in regard to HRQoL (primary outcome). Moreover, there is also a need to analyze functional performance limited by dyspnea in ADLs, the impact of COPD on patients’ lives, and these patients’ pulmonary function (secondary outcomes). We therefore propose a randomized clinical trial with the evaluators blinded in addition to blinding the researcher to patient allocation. The data will be coded in a nonidentifiable way and will not contain any information that could indicate bias regarding patient allocation. The study will have a 16-week follow-up, with reapplication of tests and questionnaires at 8 weeks, enabling assessment of the responses in the short and long term, which, according to our previous review, has not yet been performed. In addition, our control group will not undergo any intervention, thereby providing analysis of the isolated effect of acupuncture on patients with COPD.

Control procedures involving invasive or noninvasive sham needling techniques can be therapeutically active, evoking localized neurophysiological and/or immune and circulatory responses [46,47,48,49]. There are also variations in assumptions about the accuracy needed for point location, as some acupuncturists and researchers consider acupuncture points areas of reactivity rather than action points. These assumptions affect the integrity of sham acupuncture as an appropriate control [28].

The PEDro (Physiotherapy Evidence Database) provides easy access to high-quality clinical research by physical therapists around the world, enabling the application of evidence in practice and effective teaching. All randomized clinical trials indexed in the PEDro are classified and can receive a maximum score of 10 points, according to their quality through the PEDro scale. This classification enables readers to identify relevant and valid studies to guide them in clinical practice [50].

A bibliographic survey using the PEDro database shows only one study that evaluated the effect of AT on the quality of life of patients with COPD with a score of 8; the other studies had scores of 5 or less, that is, were of low methodological quality according to the database. Although Feng’s 2016 study obtained a score of 8 in terms of methodological quality, gaps remain regarding the isolated effects of acupuncture, as the study used a sham group as a comparator [22]. The use of sham acupuncture using the application of the needle at points other than those of oriental medicine or the use of seeds in place of the needle or even the use of a placebo needle can generate interpretation bias considering the mechanical effects and the pressure exerted on the biological tissue. Given the consideration that sham acupuncture can be as effective as traditional acupuncture [46,47,48,49], there remains a gap regarding the isolated effects of acupuncture. Furthermore, in addition to HRQoL, secondary outcomes such as functional performance, functional limitations, dyspnea in activities of daily living, and the impact of the disease on life are crucial for the global understanding of the effectiveness of acupuncture in patients with COPD. Therefore, conducting the present study is justified in terms of relevance and practical potential for application.

Our work predicts a score of 8 on the PEDro scale as we plan to specify eligibility criteria, randomly assign patients, and use veiled subject allocation by a researcher designated exclusively for this role during research planning, performed using a computer program; we will have well-defined inclusion and exclusion criteria ensuring similar groups with regard to the most important prognostic indicators at baseline; and there will be blinding of the evaluators who will measure the outcomes. Furthermore, we will aim to ensure patient adherence, ensuring that measurements of at least one key outcome will be obtained in more than 85% of subjects initially distributed among the groups, using weekly telephone calls made to patients in the control group; data will be analyzed by intent to treat; we will describe the results of intergroup statistical comparisons in at least one key result; and we will describe measures of precision as measures of variability for at least one key outcome. We will lose in points that do not apply to studies with acupuncture, i.e., blinding of subjects and blinding of the therapist who will apply the therapy. In addition, we will follow up for a total of 16 weeks by reapplying the tests and questionnaires at 8 weeks, which will enable assessment of the responses in the short and long term, which, according to our previous review, has not yet been undertaken.

## Data Availability

The data presented in this study are available on request from the corresponding author. The data are not publicly available due to the privacy guarantee of the data collected individually.
